# Paralysis—Cerebral, Bulbar and Spinal

**Published:** 1886-11

**Authors:** 


### Reviews.

**Paralysis—Cerebral, Bulbar and Spinal.** A Manual of Diagnosis for Students and Practitioners. By H. CHARLTON BASTIAN, M. A., M. D., F. R. S., Fellow of the Royal College of Physicians; Examiner in Medicine, at the Royal College of Physicians; Professor of Clinical Medicine and of Pathological Anatomy, in University College, London, Physician to University College Hospital and to National Hospital for Paralyzed and Epileptics; Crown Referee in cases of supposed Insanity; with numerous illustrations. New York: D. Appleton & Company, 1 3 and 5 Bond St. 1886.

This work is designed to facilitate diagnosis of the various forms of paralysis. How great was the need for just such a work every student and practitioner well knows. There is no subject concerning which there is so little knowledge, among the general practitioners, as this. The various forms of paralysis from brain disease, from disease of the bulb and from disease of the spinal cord are now so numerous, and many recent additions have been made to our knowledge in these directions, that some such aid to diagnosis may well be looked for by those for whom this work is intended. There is a chapter on Paralysis of Encephalic, spinal, peripheric, origin which adds much to the work and gives one a clear idea of the various forms to start with. At the head of many of the chapters a review of the physiology and anatomy of the various parts is to be found, which greatly aids in understanding the pathological condition. "The signs of paralysis of the different cranial nerves have been pretty fully dealt with, because the recognition of such paralysis is often a matter of the greatest importance. Concerning the importance of ariving at a correct diagnosis it is needless to dilate." The book supplies a want long felt; to come from this celebrated author makes it much more valuable.

